# Associations between a history of sexual abuse and dental anxiety, caries experience and oral hygiene status among adolescents in sub-urban South West Nigeria

**DOI:** 10.1186/s12903-021-01562-8

**Published:** 2021-04-19

**Authors:** Morenike Oluwatoyin Folayan, Maha El Tantawi, Nourhan M. Aly, Abiola Adetokunbo Adeniyi, Elizabeth Oziegbe, Olaniyi Arowolo, Michael Alade, Boladale Mapayi, Nneka Maureen Chukwumah, Olakunle Oginni, Nadia A. Sam-Agudu

**Affiliations:** 1 Department of Child Dental Health, Obafemi Awolowo University, Ile-Ife, Nigeria; 2 Department of Pediatric Dentistry and Dental Public Health, Faculty of Dentistry, Alexandria University, Alexandria, Egypt; 3 Department of Preventive Dentistry, Lagos State University College of Medicine, Ikeja, Nigeria; 4 Obafemi Awolowo University Teaching Hospitals Complex, Ile-Ife, Nigeria; 5 Department of Mental Health, Obafemi Awolowo University, Ile-Ife, Nigeria; 6 Department of Preventive Dentistry, School of Dentistry, University of Benin, Benin City, Nigeria; 7 International Research Center of Excellence, Institute of Human Virology Nigeria, Abuja, Nigeria; 8 Institute of Human Virology and Department of Pediatrics, University of Maryland School of Medicine, Baltimore, USA

**Keywords:** Sexual and reproductive health, Mental health, Oral health, Adolescents, Nigeria

## Abstract

**Introduction:**

Sexual and oral health are important areas of focus for adolescent wellbeing. We assessed for the prevalence of sexual abuse among adolescents, oral health factors associated with this history, and investigated whether sexual abuse was a risk indicator for dental anxiety, caries experience and poor oral hygiene.

**Methods:**

This was a cross-sectional study conducted between December 2018 and January 2019 among adolescents 10–19 years old in Ile-Ife, Nigeria. Survey data collected included respondents’ age, sex, and socioeconomic status, oral health risk factors (dental anxiety, frequency of tooth brushing intake of refined carbohydrates in-between-meals, flossing, dental visits, smoking, alcohol intake, use of psychoactive substances), caries experience, oral hygiene status, history of sexual abuse, and sexual risk behaviors (age of sexual debut, history of transactional sex, last sexual act with or without condom, multiple sex partners). Regression models were constructed to determine the association between outcome variables (dental anxiety, presence of caries experience and poor oral hygiene) and explanatory variables (oral health risk factors and history of sexual abuse).

**Results:**

The prevalence of sexual abuse in our cohort was 5.9%: 4.3% among males and 7.9% among females. A history of sexual abuse was associated with alcohol consumption (*p* = 0.009), cigarette smoking (*p* = 0.001), and a history of transactional sex (*p* = 0.01). High/severe dental anxiety was significantly associated with increased odds of a history of sexual abuse (AOR = 1.81; 95% CI 1.10, 2.98), but not with caries experience (AOR = 0.66; 95% CI 0.15, 2.97) nor poor oral hygiene (AOR = 1.68; 95% CI 0.95, 2.96). Dental anxiety was associated with increased odds of alcohol intake (AOR = 1.74; 95% CI 1.19, 2.56), twice daily tooth brushing (AOR = 1.48; 95% CI 1.01, 2.17) and daily consumption of refined carbohydrates in-between-meals (AOR = 2.01; 95% CI 1.60, 2.54). Caries experience was associated with increased odds of using psychoactive substances (AOR = 4.83; 95% CI 1.49, 15.62) and having low socioeconomic status (AOR = 0.40; 95% CI 0.18, 0.92). Poor oral hygiene was associated with increased odds of having middle socioeconomic status (AOR = 1.43; 95% CI 1.05, 1.93) and daily consumption of refined carbohydrates in-between-meals (AOR = 1.38; 95% CI 1.08, 1.78).

**Conclusion:**

Adolescents who are highly dentally anxious need to be screened for a history of sexual abuse to facilitate access to professional care and support.

**Supplementary Information:**

The online version contains supplementary material available at 10.1186/s12903-021-01562-8.

## Background

It is important for healthcare providers to be aware of the different ways in which sexual abuse can manifest in survivors and its impact on multiple facets of the health of survivors. The oral cavity is a common site of sexual abuse [1], with the risk for sexually transmitted oral infections such as gonorrhea and chlamydia [2, 3]. Oral and peri-oral warts caused by human papillomavirus may also be sexually transmitted [4]. Besides infections, physical damage may be evident: forced oral sex could result in petechial hemorrhage at the junction of the hard and soft palate [4]. Sexual abuse is associated with an increased risk of HIV infection, which is enhanced by deregulation of the protective immune system [5]. Sexual abuse is also associated with the survivor’s engagement in sexually risky behaviors such as having sex with multiple or concurrent partners, transactional sex, drug use before or during sex, and sex with a high-risk partner [6]. Among survivors, rape experience is also associated with low self-esteem and low competency in refusing unwanted sex, and in negotiating condom use [7, 8].

Adolescents and young people are at high risk of experiencing sexual violence [9]. In Nigeria, although both male and female adolescents report forced sex, females are disproportionately affected [10, 11]: approximately 31% of female and 6% of male adolescents in Nigeria report forced sexual initiation [12]. Sexual violence survivors in Nigeria are frequently stigmatized, with little legal, psychological, or therapeutic support [13]. Especially important but often neglected are the residual psychological impacts of sexual abuse on body image among survivors. Since body image is important for young people [14], adolescents may have increased interest in oral health care as a component strategy to improve their physical aesthetics and appeal [15]. Avoiding health services as a coping strategy among adolescents who have experienced sexual abuse [16] may therefore be counterproductive. Yet, avoidance of dental services may still occur, since dental procedures may be perceived as invasive to their privacy, and out of their control [17]. Also, post-traumatic stress disorder, low self-esteem and depression associated with sexual abuse may make survivors care less about their oral health. Dental service utilization may be associated with dental anxiety, experiences of flashbacks, feelings of embarrassment, and anxiety about physical proximity to the dentist; the sex of the dentist may also trigger memories of the perpetrator [17]. Survivors of sexual abuse may also have disproportionate dental problems [18].

The objectives of this study were to determine the prevalence of and factors associated with the history of sexual abuse among adolescents in Nigeria. In addition, we investigated whether sexual abuse was a risk indicator for dental anxiety, the presence of caries experience and poor oral hygiene.

## Methods

### Study population and study design

Data were collected through a household survey conducted between December 2018 and January 2019 in Ile-Ife, in the Ife Central Local Government Area, a semi-urban community of Osun State, South West Nigeria. Adolescents aged 10–19 years old from whom parental consent/assent/individual informed consent were obtained as appropriate, were eligible to participate. Adolescents who were critically ill and could not provide independent responses to the study survey were excluded from participation. Recruitment of participants continued until the sample size for the study was reached.

### Sample size and sampling technique

The minimal sample size was calculated with the formula proposed by Araoye [19]. With a caries prevalence of 13.9% [20] a margin of error of 5%, and a confidence level of 95%, a minimum sample size of 1323 adolescents was estimated.

Adolescents were recruited using a multi-stage sampling technique. First, 70 of the 700 enumeration areas in Ife Central Local Government Area were selected using simple random technique. Next, every other household in the selected enumeration areas was identified as an eligible household. Finally, in each eligible household, one adolescent who met inclusion criteria was recruited for study participation. Whenever a household declined to participate, the next eligible household was substituted.

### Socio-demographic profile

Information on age at last birthday, sex at birth and parents’ level of education and occupation were collected. Socioeconomic status was determined using an adapted version of the index developed by Olusanya et al. [21], which was used in a previous study in the study setting [22]. The index is a combination of the mother’s education with the father’s education and occupation. The study participants’ mother’s level of education was classified as follows: no formal education, Quranic and primary school education (score 2); secondary school education (score 1) and tertiary education (scored 0). The father’s occupation was also categorized into three levels: civil servants or skilled professionals with a tertiary level of education (score 1); civil servants or skilled professionals with a secondary level of education (score 2); unskilled, unemployed, students, and civil servants or skilled professionals with a primary and or Quranic education (scored 3).

The social class of the adolescent was determined by adding the score of the mother’s education to that of the father’s occupation. Each adolescent was allocated into social classes I–V (class I, upper class; class II, upper middle class; class III, middle class; class IV, lower middle class; class V, lower class). These categories were further collapsed into three categories namely: high (classes I and II), middle (class III) and low (classes IV and V) for analysis. When an adolescent had lost a parent, their socioeconomic status was determined using the status of the living parent. The data collection tool is attached as Additional file 1.

### History of sexual abuse

Respondents were provided a definition of sexual abuse (an unwanted sexual advancement where there was penetration of the mouth, vagina and/or anus by using force, making threats or taking advantage of you) [23]; and asked if they had had any experience of sexual abuse (yes, no, no response). The “no response” category was removed from further analysis.

### Sexual practices and sexual behavior

Participants were asked if they had ever had vaginal or anal sexual intercourse; had sex in exchange for money, a place to stay, or material goods (transactional sex); number of male and female sex partners, and if a condom was used at the last vaginal and/or anal intercourse. These measures were similar to others that have been found reliable and valid in previous research [24].

### Tooth brushing

Respondents were also asked to indicate the frequency of toothbrushing, using the following options—irregularly or never, once a week, a few (2–3) times a week; once a day, and more than once a day. Respondents who chose the options ‘irregularly or never, once a week, a few (2–3) times a week, once a day’ were classified as having undertaken poor preventive dental care [25].

### Consumption of refined carbohydrates in-between-meals

Respondents were also asked to indicate the frequency of consuming sugar-containing snacks or drinks between main meals, using the following options—about 3 times a day or more, about twice a day, about once a day, occasionally; not every day, rarely or never eat between meals. Respondents who chose the options ‘about 3 times a day or more, about twice a day, about once a day’, were classified as not having undertaken adequate preventive dental care [25].

### Use of dental floss

Respondents were also asked to indicate how often dental floss was used for cleaning their teeth, using the following options—Not at all, occasionally, a few (2–3) times a week, once in a day, more than one time in a day. Respondents, who chose the options ‘not at all, occasionally, a few (2–3) times a week’, were classified as not having undertaken adequate preventive dental care [25].

### Dental service utilization

Respondents were also asked to indicate the timing of their last dental check-up, using the following options—within the last 6 months, more than 6 months to 1 year ago, more than 1 to 2 years ago, more than 2 to 5 years ago, more than 5 years, never, do not remember. Attending a dental check-up within the last year was defined as preventive care use (categorized as ‘yes’ for the analysis). Respondents who chose the options ‘more than 1 to 2 years ago, more than 2 to 5 years ago, more than 5 years, never, do not remember’ were classified as not having undertaken adequate preventive dental care (categorized as ‘no’ for the analysis) [25].

### Dental anxiety

Dental anxiety refers to patients' specific response to dental situations associated with stress [26]. Dental anxiety was measured using the four item Corah dental anxiety scale [27]. Each item was scored on a five-point Likert scale ranging from 0 to 4. The level of anxiety was assessed as the sum of the responses to all the questions. Possible scores ranged from 0 to 16 and were coded as follows, 0 to 4 as normal anxiety, 5–8 as moderate anxiety, 9–12 as high anxiety, and 13–16 as severe anxiety bordering on phobia. The scale has been validated for use in Nigeria [28] and the Cronbach’s alpha for the present study was 0.92.

### Smoking habits

The questionnaire requested information on the respondents’ habits of cigarette smoking separately. The questions had six response options—'No, never; No, I used to, but I quit; Yes, once a month or less; Yes, a few times (2–3) a month; Yes, a few times (2–3) a week; and Yes, once a day or more. All those who chose options ‘Yes, once a month or less, Yes, a few times (2–3) a month, Yes, a few times (2–3) a week, Yes, once a day or more’ were classified as smokers. Respondents with other responses were classified as non-smokers [25].

### Intake of alcohol

Respondents were asked about their frequency of alcohol intake (Everyday, once a week, less than once a week, never, not sure and no response). The responses were categorized into Yes (Every day, once a week, less than once a week) and No (never, not sure).

### Use of psychoactive drugs

Information on the use of psychoactive drugs (marijuana, solvent glue, cocaine, heroin, tramadol, codeine, injecting cocaine or heroin using a syringe and needle) was collected. The responses were dichotomized into ‘0’ when there was no indication of psychoactive substance use, and ‘1’ if there was indication of use of any of these drugs.

### Oral hygiene

Oral hygiene status was evaluated with the simplified oral hygiene index (OHI-S) described by Greene and Vermillion [29]. The amount of debris or calculus present on the facial or lingual surfaces of six index teeth (3, 8, 14, 19, 24, and 30) in the permanent dentition was used to determine the debris and calculus index scores, with scores ranging from 0 to 3, from which the OHI-S score was calculated by adding the two scores with score ranging from 0 to 6 [30]. The oral hygiene was classified as good, fair, or poor when the scores were 0.0–1.2, 1.3–3.0, and > 3.0, respectively. Oral hygiene was further dichotomized into ‘good oral hygiene’ and ‘fair/poor oral hygiene’ for analysis.

### Caries experience

Assessment of caries experience was performed after oral hygiene and gingival health status were determined. Each participant was examined sitting on a chair under natural day light, using a dental mirror. Teeth were cleaned of debris and dried using a sterile gauze before assessment. The presence of caries experience was assessed using the decayed (D), missing (M), and filled (F) teeth (T) index following the World Health Organization criteria at cavitation level [30]. The DMFT score for an individual was the sum of the scores of the D, M and F components of the index.

### Data analysis

Descriptive analysis was conducted to determine the proportion of adolescents with each sociodemographic variable (age, sex, socioeconomic status), oral health risk factors (dental anxiety, tooth brushing frequency, flossing, frequency of intake of refined carbohydrate in-between-meals, and dental visits within the last year), the presence of caries experience and oral hygiene status. Proportions of adolescents with a history of sexual abuse, and sexual risk behaviors (age of sexual debut, history of transactional sex, last sexual act with or without condom, multiple sex partners) were also determined.

Bivariate analysis was conducted to determine the association between the explanatory variable (sexual abuse) and outcome variables (dental anxiety, the presence of caries experience and fair/poor oral hygiene) using Chi-squared and t-tests. Multivariable regression models were specified to identify risk factors independently associated with oral health factors: an ordinal logistic model was specified for levels of dental anxiety, while separate binary logistic regression models were specified for the presence of caries experience and poor oral hygiene. Confounders adjusted for in the models included sex, age, socioeconomic status, intake of alcohol, use of psychoactive substances and cigarette smoking. The explanatory variables were oral health habits (brushing twice daily, flossing and consumption of refined carbohydrates in between meals) and reporting a history of sexual abuse. The estimated coefficients, expressed as adjusted odds ratios (AOR) and their 95% confidence intervals, were calculated and reported. Statistical analyses were conducted with IBM SPSS for windows (version 23.0). Statistical significance was inferred at *p* < 0.05.

### Ethical considerations

Ethical approval was obtained from the ethics and research committee of the Institute of Public Health, Obafemi Awolowo University, Ile-Ife, Nigeria (IPHOAU/12/669). Approval for conduct of the study was obtained from the Local Government Authority prior to commencement. Informed consent was obtained from the parent of each study participant aged 10–11 years old prior to enrollment. Parental consent and participant assent were obtained for those 12–13 years old. Consent was obtained from study participants 14 to 19 years old, in line with guidance from the Federal Ministry of Health [31]. Efforts were made to minimize risks regarding participants’ loss of confidentiality by ensuring anonymized data collection was done privately and collected with an electronic data platform. Study participants’ discomfort with the personal nature of questions was limited by ensuring that field workers were trained on how to ask sensitive questions and ascertain non-verbal cues observed during interviews. No compensation was paid to surveyed adolescents for study participation.

## Results

Complete responses were available for 1056 adolescents. Post-hoc power analyses indicated the power is 0.89 to detect significant associations between sexual abuse and dental anxiety. The mean age of study participants was 14.6 years. Out of the 1,056 adolescents for whom there were complete data 598 (56.6%) were male, 383 (36.3%) were of high socioeconomic status, 103 (9.8%) reported alcohol intake, 48 (4.5%) reported use of psychoactive substances, 13 (1.2%) reported cigarette smoking, and 80 (7.6%) were sexually active and reported previously having sexual intercourse.

Among the 80 participants who reported having sexual intercourse, 47 (58.8%) had one current sex partner, 31 (38.8%) used a condom at the last sexual intercourse and 5 (6.3%) reported a history of transactional sex (Table 1).

**Table 1:** Sociodemographic and high sexual risk behavior of adolescents with and without a history of sexual abuse in Ile-Ife, Nigeria (n = 1056)

Variables	History of sexual abuse N = 62 n (%)	No history of sexual abuse N = 994 n (%)	*p* value	Total n (%)
Sex				
Male	26 (41.9%)	572 (57.5%)	0.02*	598 (56.6%)
Female	36 (58.1%)	422 (42.5%)		458 (43.4%)
Age				
Mean (SD)	16.6 (2.2)	14.4 (2.6)	< 0.001*	14.6 (2.6)
Socioeconomic status				
High	22 (35.5%)	361 (36.3%)	0.31	383 (36.3%)
Middle	16 (25.8%)	331 (33.3%)		347 (32.9%)
Low	24 (38.7%)	302 (30.4%)		326 (30.9%)
Intake of alcohol				
Yes	12 (19.4%)	91 (9.2%)	0.009*	103 (9.8%)
No	50 (80.6%)	903 (90.8%)		953 (90.2%)
Use of other psychoactive substances				
Yes	4 (6.5%)	44 (4.4%)	0.52	48 (4.5%)
No	58 (93.5%)	950 (95.6%)		1008 (95.5%)
Cigarette smoking				
Yes	5 (8.1%)	8 (0.8%)	0.001*	13 (1.2%)
No	57 (91.9%)	986 (99.2%)		1043 (98.8%)
Ever had sexual intercourse				
Yes	24 (38.7%)	56 (5.6%)	< 0.001*	80 (7.6%)
No	38 (61.3%)	938 (94.4%)		976 (92.4%)
Age of sexual debut [N = 80]				
Mean (SD)	15.30 (3.74)	15.62 (2.12)	0.65	15.53 (2.64)
Number of current sex partners [N = 80]				
0	3 (12.5%)	6 (10.7%)	0.81	9 (11.3)
1	15 (62.5%)	32 (57.1%)		47 (58.8)
> 1	6 (25.0%)	18 (32.1%)		24 (30.0)
Use of condom at last sex [N = 80]				
Yes	7 (29.2%)	24 (42.9%)	0.25	31 (38.8)
No	17 (70.8%)	32 (57.1%)		49 (61.3)
History of transactional sex [N = 80]				
Yes	4 (16.7%)	1 (1.8%)	0.01*	5 (6.3%)
No	20 (83.3)	55 (98.2%)		75 (93.8%)

*Statistically significant at *p* < 0.05

The prevalence of sexual abuse in our cohort was 5.9%: 4.3% among males and 7.9% among females. Table 1 shows that a greater percentage of adolescents who reported a history of sexual abuse compared to those who did not, were female (58.1% vs 42.5%; *p* = 0.02), older (16.6 years vs 14.4 years; *p* < 0.001), consumed alcohol (19.4% vs 9.2%; *p* = 0.009), smoked cigarettes (8.1% vs 0.8%; *p* = 0.001), had a history of sexual intercourse (38.7% vs 5.6%; *p* < 0.001) and reported a history of transactional sex (16.7% vs 1.8%; *p* = 0.01).

Table 2 presents oral health profile of adolescents with and without a history of sexual abuse. Overall, only 92 (8.7%) adolescents brushed their teeth at least twice daily, 630 (59.7%) consumed refined carbohydrates in-between-meals more than once daily, 123 (11.6%) used dental floss daily, and 13 (1.2%) visited the dentist in the last 12 months. The prevalence of severe dental anxiety was 20.6%, 39 (3.7%) had caries experience, and 55.4% had fair/poor oral hygiene. A greater percentage of those with normal levels of dental anxiety had a history of sexual abuse (45.2% vs 27.6%; *p* = 0.01).

**Table 2:** Oral health behavior of adolescents with and without a history of sexual abuse among a cohort in Ile-Ife, Nigeria [N = 1,056]

Variables	History of sexual abuse N = 62 n (%)	No history of sexual abuse N = 994 n (%)	*p* value	Total n (%)
Tooth brushing				
≤ 1 time a day	59 (95.2%)	905 (91.0%)	0.36	964 (91.3%)
≥ 2 times a day	3 (4.8%)	89 (9.0%)		92 (8.7%)
Consumption of refined carbohydrates in-between-meals				
< 1 time a day	19 (30.6%)	407 (40.9%)	0.11	426 (40.3%)
≥ 1 time a day	43 (69.4%)	587 (59.1%)		630 (59.7%)
Daily use of dental floss				
Yes	8 (12.9%)	115 (11.6%)	0.75	123 (11.6%)
No	54 (87.1%)	879 (88.4%)		933 (88.4%)
Visited the dentist in the last 12 months				
Yes	0 (0%)	13 (1.3%)	1.00	13 (1.2%)
No	62 (100%)	981 (98.7%)		1043 (98.8%)
Dental anxiety				
Normal	28 (45.2)	274 (27.6)	0.01*	302 (28.6)
Moderate	20 (32.3)	374 (37.6)		394 (37.3)
High	8 (12.9)	134 (13.5)		142 (13.4)
Severe	6 (9.7)	212 (21.3)		218 (20.6)
Oral hygiene				
Good	21 (33.9)	450 (45.3)	0.08	471 (44.6)
Fair/ poor	41 (66.1)	544 (54.7)		585 (55.4)
Caries experience				
Present	2 (3.2%)	37 (3.7%)	1.00	39 (3.7%)
Absent	60 (96.8%)	957 (96.3%)		1017 (96.3%)

X^2^, Fisher exact and t-test were used

*Statistically significant at *p* value < 0.05

Table 3 shows the results of the tests of association between the history of sexual abuse and dental anxiety levels, the presence of caries experience and poor oral hygiene. The odds of having high/severe dental anxiety were significantly higher for adolescents who reported a history of sexual abuse (AOR = 1.81, 95% CI 1.10, 2.98) when compared with adolescents who did not have such a history; higher for adolescents who reported alcohol intake (AOR = 1.74, 95% CI 1.19, 2.56) when compared with those reporting no alcohol intake. The odds of high/severe anxiety was also higher for adolescents who reported twice daily tooth brushing (AOR = 1.48, 95% CI 1.01, 2.17) when compared with those reporting once or no daily tooth brushing; and for adolescents who reported daily consumption of carbohydrates in-between-meals (AOR = 2.01, 95% CI 1.60, 2.54) when compared with adolescents who did not report such.

**Table 3:** Regression analysis to determine association between history of sexual abuse and levels of dental anxiety, the presence of caries experience and poor oral hygiene among adolescents in Ile-Ife, Nigeria [N = 1056]

	High/severe dental anxiety AOR (95% CI)	Presence of caries experience AOR (95% CI)	Poor oral hygiene AOR (95% CI)
Gender (male vs female)	1.24 (0.99, 1.54)	0.91 (0.47, 1.75)	1.21 (0.95, 1.55)
Age	1.02 (0.98, 1.06)	1.13 (0.99, 1.29)	0.99 (0.95, 1.04)
Socioeconomic status			
High versus low	0.93 (0.71, 1.23)	0.40 (0.18, 0.92)*	0.78 (0.57, 1.05)
Middle versus low	1.26 (0.96, 1.65)	0.53 (0.24, 1.15)	1.43 (1.05, 1.93)*
Consumption of alcohol intake Reference: No	1.74 (1.19, 2.56)*	0.61 (0.19, 1.93)	1.19 (0.78, 1.83)
Use psychoactive drugs use Reference: No	1.55 (0.88, 2.73)	4.83 (1.49, 15.62)*	0.63 (0.33, 1.19)
Cigarette smoking Reference: No	0.64 (0.24, 1.71)	0.77 (0.08, 7.48)	0.99 (0.29, 3.35)
Tooth brushing at least twice daily Reference: No	1.48 (1.01, 2.17)*	0.84 (0.25, 2.83)	1.37 (0.89, 2.10)
Daily flossing Reference: No	0.93 (0.68, 1.26)	1.16 (0.50, 2.71)	1.10 (0.79, 1.55)
Daily consumption of refine carbohydrates in-between-meals Reference: No	2.01 (1.60, 2.54)*	0.76 (0.39, 1.49)	1.38 (1.08, 1.78)*
Sexual abuse Reference: No	1.81 (1.10, 2.98)*	0.66 (0.15, 2.97)	1.68 (0.95, 2.96)

*AOR* adjusted odds ratio, *CI* confidence interval

*Statistically significant at *p* value < 0.05

The odds of having caries experience was significantly higher for adolescents who used psychoactive substances (AOR = 4.83, 95% CI 1.49, 15.62) when compared with those who did not; and significantly lower for adolescents who had high socio-economic status when compared with those with low socioeconomic status (AOR = 0.40, 95% CI 0.18, 0.92).

There were significantly higher odds of poor oral hygiene for participants with middle socioeconomic status than those from low socioeconomic status (AOR = 1.43, 95% CI 1.05, 1.93) and for those who consumed refined carbohydrates in-between-meals daily (OR = 1.38, 95% CI 1.08, 1.78).

## Discussion

In our Nigerian adolescent cohort, the prevalence of a history of sexual abuse was higher for females than males and was associated with risky behaviors such as alcohol intake, cigarette smoking, and transactional sex. A history of sexual abuse was independently associated with higher levels of dental anxiety, but not with the presence of caries experience nor poor oral hygiene. The presence of caries experience was associated with low socioeconomic status and the use of psychoactive substances. Oral hygiene was significantly worse for those with middle socioeconomic status and for those who reported daily consumption of refined carbohydrates in-between-meals.

One of the strengths of this study is the data collection method—a household survey was conducted, making it possible to determine the population level prevalence of sexual abuse. The findings can thus be generalized to populations with epidemiological profiles similar to those of the study setting. Despite this strength, the study has a few limitations. First, the diagnosis of caries was limited to clinical examination; the non-use of radiographic techniques may have led to underestimation of the prevalence. Second is the risk of recall bias. Although childhood experiences can be recalled accurately [32], there is a risk of under-reporting sexual abuse and other sexual history due to social desirability bias, especially in a culture that is sexually conservative [33]. Under reporting of sexual abuse is a threat because it dilutes the strength of the association [34, 35]. We attempted to reduce this risk by training data collectors on how to collect sensitive data and engaging young data collectors to enable respondents relate with their peers. This was because we were aware that under-reporting was also associated with interviewer characteristics [36]. The questionnaire was also intentionally designed to minimize respondents feeling threatened or judged by their sexual history. Thirdly, being a cross-sectional study, it is not possible to establish a cause–effect relationship from the study findings. The study findings, however, represent the first evidence on sexual abuse and oral health in Nigerian adolescents. This has resulted in the development of new hypotheses that can be further explored through future studies on relationships between sexual abuse and oral health in adolescents.

The prevalence of sexual abuse we reported is lower than the 25.7% reported among 10–19-year-old adolescents in Lagos State—a cosmopolitan settlement also located in South-West Nigeria [37]. The disparity in our prevalence estimates may reflect the different settings in which the studies were conducted. Our study was carried out in a semi-urban setting, while the Lagos study was carried out in a densely populated urban community, which may be characterized by higher levels of crime and socioeconomic inequalities. However, our findings are closer to the prevalence of 5% reported for sub-Saharan Africa [38] and falls within the range of 8 to 31% reported for girls and 3 to 17% reported for boys, globally [39]. A prior study indicated that the highest global prevalence of sexual abuse among adolescents was in Africa [40], making it important to study and identify risk indicators for sexual abuse among youth on the continent. The differing prevalence between the type of residential locations in Nigeria suggests that the residential location may be a risk factor for sexual abuse. Past studies had indicated that sexual abuse of young people was more prevalent in rural than urban communities [41]. This study was unable to determine differences in the prevalence between residential location; and we noticed no difference in the prevalence by socioeconomic status, a factor that also determines residential location [42].

A history of sexual abuse in childhood may lead to multiple complications, which range from immediate psychological consequences to chronic effects that can affect adjustment throughout development into adulthood [43–45]. Mental health consequences in adulthood include post-traumatic stress disorder, substance abuse disorders (drugs or alcohol), personality disorders [46]—and dental anxiety [47–49]—as abuse survivors find dental attendance very stressful [50]. Additionally, the association between sexual abuse and use of psychoactive substances (alcohol and cigarettes) may reflect the use of maladaptive coping strategies. Alternatively, it is possible that sexual abuse experiences occur in the context of substance abuse, although this was not specifically investigated in our study. Interestingly, we found an association between sexual abuse and transaction sex; a finding that was also reported in a population of adolescents in Nigeria, with suggestions that this association may be linked to low self-esteem and vulnerability to depression [51]. It is important to explore this possible link in future studies, to be able to identify if a history of transactional sex among adolescents may be a sign or manifestation of depression.

If dental anxiety keeps adolescents with a history of sexual abuse away from the dental clinic, the risks for periodontal disease and dental caries is increased [52]. However, our results do not corroborate prior findings of associations between sexual abuse and increased risk for caries, as we found no such association between sexual abuse and caries experience or poor oral hygiene. Past studies conducted in resource-rich countries have linked associations between sexual abuse and poor oral health to poor dental clinic attendance for preventive dental care stemming from dental anxiety [16]. Past studies conducted in Nigeria had indicated that dental service utilization is typically for curative and not preventive dental care [53]. For this reason, a history of sexual abuse may not be a distinctive risk factor for poor oral health in the study environment, as may be found in other settings where preventive dental service utilization is a protective factor from poor oral health.

High dental anxiety and intake of alcohol, twice or more daily toothbrushing and daily intake of refined carbohydrates in-between-meals may be connected. Patients with a high level of dental anxiety are more prone to having a high level of comorbid psychiatric disorders and symptoms [54]. Some psychiatric disorders are associated with increased intake of alcohol [55] and high consumption of refined carbohydrates [56], while others, such as obsessive–compulsive disorder, may be associated with high frequency of tooth brushing [57]. Psychiatric disorders are major morbidity problems for adolescents all over the world. Our findings suggest that adolescents who have high dental anxiety, report alcohol consumption, twice or more daily tooth brushing, and daily intake of refined carbohydrates in-between-meals, should have mental health screening. It is also possible that individuals who more frequently consume refined carbohydrates and alcohol have developed more oral problems, have been exposed to oral health services, brush more frequently, and have more experiences to trigger dental anxiety. These possibilities highlight the limitation of cross-sectional designs in fully disentangling the nature of these relationships. These findings, however, highlight the need to plan for care delivery approaches for adolescents that may facilitate early diagnosis and management of psychiatric disorders and sexual abuse. Further studies are needed to explore the relationships between oral health and sexual and mental health risk indicators.

The association between sexual abuse and alcohol intake, cigarette smoking, transactional sex and poor oral hygiene may indicate increased physical, oral and sexual risk-taking behaviors among adolescents with a history of sexual abuse [58]. These risk behaviors are associated with longer-term poor health outcomes like cancer [59], periodontal disease-associated general health problems [60]; HIV infection, a risk outcome of transactional sex which is often poorly negotiated by adolescents [61–63]. When adolescents and young persons are diagnosed with these health problems, it may be important to screen for a history of sexual abuse. The results of this screening study suggest the need to investigate longer-term health impacts of sexual abuse.

The association we have reported between caries experience and low socioeconomic status is not new. Multiple studies have highlighted that caries is a disease of inequality, with individuals of low socioeconomic status and financial resources being prone to consuming high-sugar content food [64, 65]. Similarly, the use of psychoactive substances has long been associated with caries experience, due to decrease in saliva secretion, poor oral hygiene and high sugar intake [66]. We provide the first evidence of an association between the use of psychoactive substances and caries in Nigeria. The use of psychoactive substances is becoming an issue of public health concern in the country [67]. This finding suggests the need to screen for psychoactive substance use among adolescents who present with caries in dental clinics, and specific screening for methamphetamine use in patients who present with poor oral hygiene and high daily consumption of refined carbohydrates in-between-meals [65].

In conclusion, this study indicates that the prevalence of a history of sexual abuse among both male and female adolescents in sub-urban Nigeria, is similar to that reported for sub-Saharan Africa and globally. Sexual abuse is a risk indicator for dental anxiety, but not for the presence of caries experience, or for poor oral hygiene in our study population. This study’s findings suggest that screening for a history of sexual abuse among adolescents who present with high/severe dental anxiety; and screening for psychoactive substance use among those with caries may be of high value in identifying adolescents who can benefit from clinical and psychological interventions, thereby reducing the risk for long-term sequelae. Future longitudinal studies are needed to investigate for causal relationships between these identified variables, towards providing evidence for integrated oral, mental, sexual and reproductive health services for adolescents in Nigeria and globally.

## Supplementary Information

**Additional file 1**. Data collection tool on oral, mental, sexual and reproductive health of adolescents in Nigeria.

## Data Availability

All data generated for this study are presented in the manuscript. Patient-level data can however be accessible on reasonable request from the one of the authors, Morenike Oluwatoyin Folayan.

## Abbreviations

AOR: Adjusted odds ratio
CI: Confidence interval
DMFT: Decay, missing, filled tooth
HIV: Human immunodeficiency virus
OHI-S: Simplified oral hygiene index
